# Dynamic evaluation of the glycolytic determinants LDH-A and GLUT-1 enhances prognostic significance and their inhibition affects the growth of mesothelioma spheroids

**DOI:** 10.1016/j.molmet.2026.102356

**Published:** 2026-03-23

**Authors:** Marika A. Franczak, Federica Borea, Valentina Donati, Marta Aliprandi, Amir Avan, Valentina Doldi, Giulia Bononi, Kin Yip Tam, Carlotta Granchi, Ryszard T. Smolenski, Paolo A. Zucali, Filippo Minutolo, Godefridus J. Peters, Elisa Giovannetti

**Affiliations:** 1 Department of Biochemistry, Medical University of Gdansk, Poland; 2 Department of Medical Oncology, Amsterdam University Medical Centers, location VUmc, Cancer Center Amsterdam, the Netherlands; 3 IRCCS Humanitas Research Hospital, Humanitas Cancer Center, Rozzano, Milan, Italy; 4 Unit of Pathological Anatomy 2, Azienda Ospedaliero-Universitaria Pisana, 56100 Pisa, Italy; 5 Metabolic Syndrome Research Center, Mashhad University of Medical Sciences, Mashhad, Iran; 6 Molecular Pharmacology Unit, Fondazione IRCCS Istituto Nazionale dei Tumori, Milano, Italy; 7 Dipartimento di Farmacia, Università di Pisa, Pisa, Italy; 8 Faculty of Health Sciences, University of Macau, Taipa, Macau; 9 Fondazione Pisana per la Scienza, Pisa, Italy

**Keywords:** Malignant mesothelioma, Glucose transporter-1, Lactate dehydrogenase-A, Chemoresistance, New inhibitors of glycolytic metabolism

## Abstract

Energy metabolism plays a crucial role in determining the aggressiveness of cancer. In this study, we assessed the impact of drug-induced modulation on the expression and prognostic significance of crucial factors involved in glycolytic metabolism: lactate dehydrogenase A (LDH-A) and glucose transporter type 1 (GLUT-1). In patient samples diagnosed with pleural Malignant Mesothelioma (MM), expression levels of LDH-A and GLUT-1 were studied both at baseline and after platinum-based-chemotherapy. High GLUT-1 and LDH-A levels were associated with shorter survival, and chemotherapy increased GLUT-1 expression, further correlating with poor prognosis. Utilizing LDH-A (NHI-2) and GLUT-1 (PGL14) inhibitors, we examined their effects on migration and apoptosis in immortalized (H2052, H2452) and primary (STO, MESO-II) MM cells. PGL14 and NHI-2 decreased migration, increased reactive oxygen species (ROS) and apoptosis rates. Inhibitors, both single and in combination, disintegrated the MM spheroids, while the bioluminescence from spheroid-forming cells decreased from 1.3 × 10^5^ in the control group to 9.7 × 10^4^ and 7.1 × 10^4^ [RLU/s] after NHI-2 and PGL14/NHI-2 treatment, respectively. Overexpression and chemotherapy-induced modulation of LDH-A and GLUT-1 correlated with poor MM prognosis. Combined inhibition of these two metabolic determinants impeded MM cell migration, stimulated ROS production and apoptosis, and affected spheroids’ growth, offering promise for new treatment development.

## Highlights

• Elevated LDH-A and GLUT-1 levels in pleural MM correlate with poor clinical outcomes.
• GLUT-1 expression increased in patients after treatment with chemotherapy.
• New inhibitors of GLUT-1 and LDH-A are effective against MM cells.
• The potential of targeting GLUT-1 and LDH-A for MM treatment.

## Graphical abstract

**Figure:**
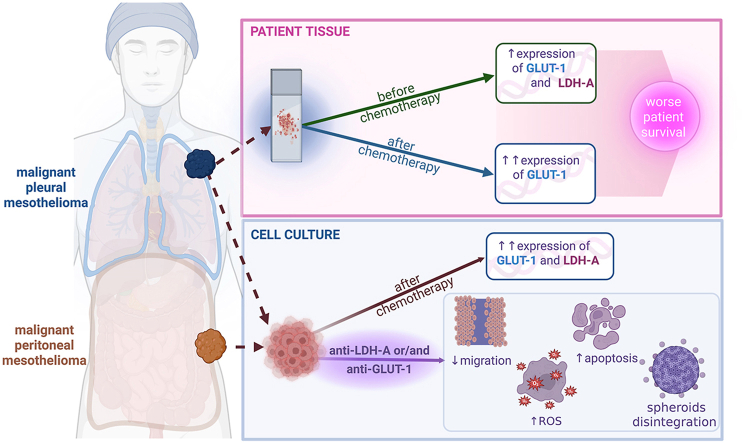

## 1 Introduction

The Warburg effect is a widely known process in which cancer cells produce energy through anaerobic glycolysis, even in the presence of oxygen. This process has been identified as one of the factors that lead to cancer aggressiveness [1]. Due to the Warburg effect, the required energy production, amino acids and lipids biosynthesis are ensured in cancer cells. Lactate, produced during aerobic glycolysis, serves as an energy product for cancer cells stimulating tumor growth and leading to extracellular acidification. These changes, among other factors, contribute to tumor progression, resistance and metastasis [2].

One of the reversible reactions of glycolysis is the conversion of pyruvate to lactate, catalyzed by lactate dehydrogenase-A (LDH-A) in the presence of nicotinamide adenine dinucleotide (NAD) as a cofactor. LDH-A plays a pivotal role in tumor malignancy by promoting invasion, angiogenesis, cell proliferation, and immune evasion [3,4]. LDH-A is highly expressed and linked to shorter survival in various cancers such as pancreatic and thoracic cancers, including mesothelioma [3].

Another key role in glycolytic metabolism is played by glucose transporters (GLUTs), a group of proteins that carry glucose molecules across cell membranes to fuel energy production. Fast proliferating cells that rely on ATP produced in aerobic or anaerobic glycolysis, like endothelial and cancer cells, have high expression of GLUT-1 to meet the substrate requirements of glucose [5]. Similar to LDH-A, in some cancers, like breast, pancreatic and colorectal cancers, a high level of GLUT-1 is often found, and associated with poor patient prognosis, aggressiveness and resistance to therapy [[6], [7], [8]]. Moreover, GLUT-1 can prompt chemotherapy resistance by itself or by promoting other signaling pathways such as YAP and AKT/mTOR pathway, affecting apoptosis induction [5].

Malignant mesothelioma (MM) is a rare and aggressive cancer that affects the pleural, peritoneal, pericardial and testicular tissues. Its development is strongly linked to asbestos exposure, and because of the late-stage diagnosis, which is usually 4 months after symptoms, and the inefficiency of standard therapy, it has a poor survival rate [9]. Histologically, MM is divided into three subtypes: epithelioid (more than 50% of cases, with the best prognosis), sarcomatoid (about 10%, with the worst prognosis) and biphasic. Since 2004, the recommended standard first-line systemic treatment for unresectable malignant mesothelioma has been a combination of platinum agents and pemetrexed (PMX), while gemcitabine (GEM) has been used as a second-line treatment [10,11]. A Phase III study showed significantly longer survival for patients with pleural MM treated with PMX and cisplatin (12.1 months) compared to cisplatin alone (9.3 months) [12]. More recently, the introduction of immune checkpoint inhibitors has led to modest improvements [13], resulting in FDA approval of the combination of ipilimumab and nivolumab. However, these benefits appear to be limited to patients with non-epithelioid histologies and are associated with a notable increase in serious adverse events and treatment discontinuation.

Thus, substantial efforts are still required to improve MM prognosis. This includes identifying new biological markers of the disease's aggressive nature and exploring novel therapeutic strategies. In line with this, we conducted our study in epithelial cell lines of MM to investigate whether there are changes in GLUT-1 and LDH-A expression following standard treatment, as we observed significant changes in MM cells treated with GLUT-1 and LDH-A inhibitors in our previous research [14,15].

It is worth mentioning that a high expression of GLUT-1 is specific to pleural MM and distinguishes it from reactive mesothelial cells, thereby offering diagnostic utility [16]. Equally significant are our prior discoveries indicating that 1) elevated levels of LDH-A correlate with diminished survival rates among patients with both pleural and peritoneal MM [15] and 2) inhibition of both LDH-A and GLUT-1 was effective in disrupting energy balance in MM cells [14]. For this purpose, we used new inhibitors of LDH-A, such as N-hydroxyindole-2-carboxylates (NHI-2) [17] and GLUT-1, i.e. the salicylketoxime derivative PGL14 [18]. Besides, we observed that hypoxia prompted increased expression of LDH-A and resistance to GEM in MM cells. However, this resistance was mitigated upon treatment with the LDH-A inhibitor NHI-1 [19].

In our previous study, we analyzed the intracellular balance of nucleotides [14]. We observed an increased NADH/NAD ratio, especially in pleural malignant mesothelioma cells, indicating LDH-A inhibition. Additionally, we observed a significant reduction in glycolysis following GLUT-1 inhibition compared to the non-treated control, confirming the inhibition of glucose entry into cells. These functional data prompted further analyses. In this study, we aimed to further advance our investigation by exploring the potential association of both LDH-A and GLUT-1 with MM prognosis, while also assessing GLUT-1 modulation in MM tissues following standard chemotherapy treatment. Furthermore, we examined the impact of combining LDH-A and GLUT-1 inhibition on various aspects of MM, including cell migration, apoptosis, reactive oxygen species (ROS) production, and spheroids’ structure.

## 2 Materials and methods

### 2.1 Immunohistochemical analysis in patients’ tissue

Previous studies, including our own, demonstrated that pleural MM is a hypoxic malignancy, as assessed by immunohistochemistry (IHC) of carbonic anhydrase IX (CAIX) [15,20]. Further, IHC studies were performed in order to assess the expression levels of LDH-A using tissue microarrays (TMAs) containing samples from 33 pleural MM patients before treatment [15], with additional GLUT-1 staining.

An additional analysis was conducted to evaluate whether chemotherapy affects LDH-A and GLUT-1 levels and its potential impact on overall survival. Tissue samples from 24 eligible pleural MM patients, treated with platinum-based compounds and PMX followed by surgery, were reviewed from the Thoracic Surgery and Oncology Department of Humanitas Research Hospital (Rozzano, Milan, Italy; 2004–2020). This study was conducted in accordance with an observational protocol that received approval from the local ethics committee of Humanitas Research Hospital (ClinicalTrials.gov NCT00867711).

The IHC analysis of LDH-A was performed as described previously [15], while IHC of GLUT-1 was performed with the commercially available monoclonal rabbit antibody, clone EP141 (Epitomics/Cellmarque, USA) at a 1:300 dilution. A staining protocol used on the machine was standardized to work equivalently on formalin-fixed tissues. Appropriate positive (red blood cells) and negative controls (no antibody) were used. The area of GLUT-1 staining was evaluated on a sliding scale of 0–3+ to represent the percentage of positive tumor cells (H-score, 0 = null, 1 = low, 2 = intermediate, 3 = high expression).

The mean value of GLUT-1 score pre- and post-chemotherapy was compared. Moreover, for each patient, the variation in the expression of GLUT-1 was calculated and expressed as a delta value. Complete clinical data and survival data were collected for the selected patients, who were then classified into two groups according to the delta value (0 versus +1 or +2).

### 2.2 Cell culture

The human pleural immortalized MM cell lines (NCI–H2052, NCI–H2452) were obtained from ATCC (Manassas, USA) and cultured in RPMI 1640 medium with l-glutamine (Merck, Germany). Additional studies were performed in human primary peritoneal MM cells (MESO-II and STO) derived from samples of patients who underwent surgery and provided by Dr. Zaffaroni (Molecular Pharmacology Unit, Fondazione IRCCS Istituto Nazionale dei Tumori, Milano, Italy) [15]. MESO-II and STO were cultured in Dulbecco's Modified Eagle's Medium F12 with 1 g/L glucose (Gibco, USA). Both media were supplemented with 10% Fetal Bovine Serum (FBS; Gibco, ThermoFisher Scientific, USA) and 1% penicillin/streptomycin (Merck, Germany). The cells were cultured at 37 °C, 5% CO_2_.

### 2.3 Modulation of gene and protein expression in cell lines

H2052 cells were plated at a 6-well plate at a density of 150,000 cells/well and incubated for 24 h with previously evaluated concentrations inhibiting 50% of cell growth (IC_50_) of GEM (55.1 nM), cisplatin (4.8 μM) and PMX (0.07 μM), alone and in combination [14,21]. To determine gene expression of LDH-A and GLUT-1, qRT-PCR reactions were performed as described earlier [22]. To evaluate the protein expression of GLUT-1 by immunocytochemistry. Cells were seeded onto glass coverslips and allowed to adhere overnight. Following treatment, cells were fixed with 4% paraformaldehyde for 15 min at room temperature and permeabilized with 0.1% Triton X-100 for 10 min. Non-specific binding was blocked with 5% bovine serum albumin for 1 h. Cells were then incubated with the primary anti–GLUT-1 antibody (1:300) overnight at 4 °C, followed by incubation with a horseradish peroxidase (HRP)-conjugated secondary antibody for 1 h at room temperature. Staining was visualized using 3,3′-diaminobenzidine, when coverslips were mounted and imaged under a bright-field microscope with identical settings for all samples.

### 2.4 LDH-A and GLUT-1 inhibitors

LDH-A inhibitor (NHI-2) and GLUT-1 inhibitor (PGL14) were synthesized in the Department of Pharmacy, University of Pisa, as previously described [17,23] and dissolved in dimethylsulfoxide (DMSO; with a final concentration after diluting these inhibitors for the treatment of the cells which was always below 0.5%, and was not inhibiting cell growth in the “control” cells treated with this vehicle). The concentrations of NHI-2 and PGL14 inhibitors that resulted in 50% growth inhibition (IC_50_) for MM cells (Table S1) were determined using the sulforhodamine B (SRB) assay, as described in our previous studies [14] and were applied in all experiment. These data showed that using a 2 × IC_50_ concentration resulted in approximately 75% growth inhibition due to the non-linear relationship typical of sinusoidal curves.

### 2.5 Migration

MM cells were placed into each well of a 96-well plate at a concentration of 50,000 cells per well. After cell attachment, a wound was created using a 96-pin scratcher. Subsequently, solutions containing inhibitors, either alone or in combination, were introduced. The closure of the wound was monitored at specified time points (0, 4, 8, 20, and 24 h) by capturing images using a Leica migration station (Leica Microsystems, Germany). These images were then subjected to analysis using Scratch Assay 6.2 software (Digital Cell Imaging Labs, Keerbergen, Belgium) to quantify differences in pixel intensity within the wound tracks across each time point.

### 2.6 Apoptosis and ROS production

A total of 10,000 cells per well (MESO-II and H2052) were seeded into a 96-well plate. The cells were then treated with LDH-A and GLUT-1 inhibitors and incubated for 48 h. Camptothecin (10 μM) was used as a positive control. Apoptosis induction was assessed using a FITC-labeled Annexin V antibody (Apoptest™, VPS Diagnostic, Hoeven, the Netherlands) along with a Binding Buffer. ROS production was evaluated using the ROS Detection Cell-Based Assay Kit (Cayman Chemical, Ann Arbor, USA) following the manufacturer's instructions, utilizing 2,7-Dichlorofluorescin Diacetate (DCFDA) at a concentration of 10 μM. Pyocyanin was used as a positive and N-acetyl cysteine as a negative control. Signal readings from the plates were obtained using a BioTek plate reader (BioTek® Instruments, Vermont, USA) and normalized to cell protein.

### 2.7 Spheroids

To create tumor 3-dimensional (3D) spheroids, 10,000 MM cells (H2052, H2452, and STO) per well were seeded onto a 96-well plate coated with 1.5% agarose. Unfortunately, we were unable to generate appropriate spheroids from MESO-II cells. Once the spheroids reached the desired size and density, they underwent treatment every other day for 6 days with the IC_50_ concentrations of LDH-A and GLUT-1 inhibitors. The changes in spheroid size and aggregation were monitored by capturing images using an AxioObserver 7 FL microscope (Carl Zeiss Microscopy GmbH, Germany), and subsequently analyzed using ImageJ software, developed by the National Institutes of Health (NIH, Bethesda, USA), measuring the area of the spheroids. Changes in spheroid area were calculated as the percentage of the spheroid surface area measured after six days of treatment (day 6) relative to the area at the beginning of treatment (day 0). Additionally, at the conclusion of the experiment, the number of cells involved in spheroid formation was determined using bioluminescence by Firefly-luciferase (F-luc) activity assessment in the STO cells, which were transduced with Fm and GC vectors according to previously established methods [24,25].

### 2.8 Statistics

The experiments were conducted at least in triplicate and repeated three times. The results are presented as average values with a standard error of the mean (SEM). To analyze the statistical significance, one-way or two-way ANOVA with post hoc tests was performed using GraphPad Prism version 9 (Intuitive Software for Science, San Diego, USA). The Kaplan-Meyer method was used to estimate overall survival (OS) in the two groups and the log-rank test was used to compare the survival curves, using SPSS software Version 24 (IBM-SPSS, Chicago, IL).

## 3 Results

### 3.1 LDH-A and GLUT-1 overexpression identify a subgroup of MM patients with worse prognosis

**Figure 1:**
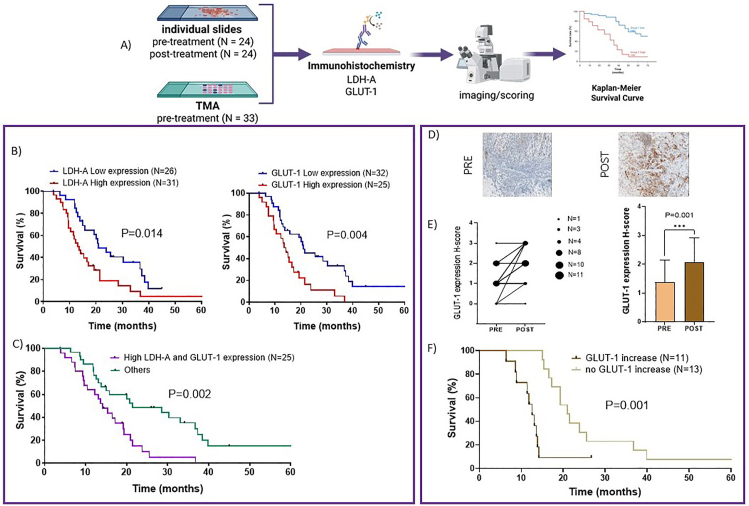
**Immunohistochemical analysis in patients' tissue.** Representative workflow (A) outlining the experiments conducted on pleural malignant mesothelioma (MM) tissues, which include immunohistochemistry (IHC) staining, imaging/scoring, and correlation with clinical outcomes. These experiments were performed on tissues collected in a tissue microarray (TMA, N = 33, pre-treatment) and using individual slides (N = 24, pre- and post-treatment). **Left box: The analysis of tissues before treatment.** (B) Kaplan–Meier curves of overall survival (OS) in pleural MM patients according to the baseline LDH-A (left panel) and GLUT-1 (right panel) high vs. low expression levels, as described above. P-values were determined with the Log-rank test. (C) OS curves according to IHC data of both LDH-A and GLUT-1 protein levels. The curves were compared using the log-rank test. **Right box: The analysis of chemotherapy (carbo/cisplatin with pemetrexed) influence on GLUT-1 expression**. (D) Representative pictures of the cores obtained from paraffin-embedded tumor material incubated with an anti-GLUT-1 specific antibody, as described in the methods; an example of weak (upper left panel) and strong staining (upper right panel) in tissues collected pre- and post-treatment (original magnification 20 × ). The area of GLUT-1 staining was scored on a semi-quantitative scale from 0 to 3+, representing the percentage of positive tumor cells. The variation in GLUT-1 expression was then calculated and expressed as a delta value to define the cut-off. (E) GLUT-1 expression showed an increase after treatment in 11 out of 24 patients, as reported in the point and bar graphs. Results are presented as mean ± SEM, compared to pre-treatment; statistical analysis was performed with two-way ANOVA with Holm-Sidak post hoc test. (F) OS curves in patients grouped according to the modulation of GLUT-1 protein levels. The curves were compared using the log-rank test.

The protein expression levels of LDH-A and GLUT-1 were evaluated using IHC on TMA and individual slides, incorporating tumor samples from a total of 57 pleural MM patients (Figure 1A). IHC staining was independently assessed by two blinded researchers; any scoring discrepancy was resolved through consensus. The concordance rate for scores from different paraffin cores of the same tumor exceeded 95%. Our previous studies [15] revealed a significant correlation between high expression levels of LDH-A and shorter survival. However, several studies have highlighted the prognostic significance of GLUT-1 protein expression across various tumor types [26]. Since data regarding MM tissues are lacking, our current study expands IHC analyses to include GLUT-1.

These analyses showed variable LDH-A and GLUT-1 protein expression among specimens, ranging from a few scattered positive cells with a weak staining to tissues with diffuse and strong cytoplasmic and membrane reactivity, in some cases accompanied by nuclear expression. In this study, 31 (54%) and 25 (44%) out of the 57 MM cases displayed high LDH-A and GLUT-1 expression, respectively.

Complete clinical data of the patients were collected, including the survival data, ranging from 6 to 78 months, with the main features summarized in Table 1. In agreement with our previous results [15], we found a significant correlation between high expression levels of LDH-A and significantly shorter OS (P = 0.014). Patients with high LDH-A expression had a median OS of 13.1 months (95% CI, 9.9–16.3), whereas patients with low expression levels of LDH-A had a median OS of 20.9 months (95% CI, 15.0–26.8). Similar results were observed when grouping the patients according to the expression levels of GLUT-1 (Figure 1B), with patients with high expression levels of GLUT-1 showing a significantly shorter OS (13.6 months (95% CI, 10.3–16.8, P = 0.004) compared to patients with low expression levels of GLUT-1 (20.9 months, 95% CI, 14.0–27.8).

**Table 1:** Pleural MM patients’ baseline characteristics.

	N = 57
Age, years [average, range]	65 [45–80]
Age	
≤65	30
>65	27
Gender	
Male	37
Female	20
Histologic subtype	
Epithelial	52
Non-epithelial	5
OS, months (median, 95%CI)	15.3 (10.0–20.7)

OS: overall survival.

Although the statistical analyses were performed on small cohorts of patients, we performed a subgroup analysis on patients with both high LDH-A and GLUT-1 protein levels (25 out of 57 patients). This subgroup exhibited a statistically significantly shorter overall survival (P = 0.002) compared to the other patients (14.2 vs. 28.5 months, Figure 1C).

### 3.2 GLUT-1 expression in pleural MM patients’ tissue increases after treatment with platinum compounds and pemetrexed

To determine the impact of chemotherapy with platinum compounds and PMX on LDH-A and GLUT-1 expression, patients' tissues were examined before and after treatment.

Interestingly, we observed a modulation of LDH-A expression in only 3 patients, precluding further statistical analyses, while in 11 out of 24 patients (46%) we observed an increase of GLUT-1 levels. Indeed, the comparison of the mean H-score value of GLUT-1 pre- and post-chemotherapy showed a statistically significant increase (p < 0.001, Figure 1D and 1E).

By grouping the patients according to the delta value of GLUT-1 before and after chemotherapy (0 vs. 1 or 2), we also observe a statistically significant difference in survival of the two groups (p = 0.001, Figure 1F).

### 3.3 GLUT-1 and LDH-A expression increases in MM cells after treatment with standard chemotherapy

**Figure 2:**
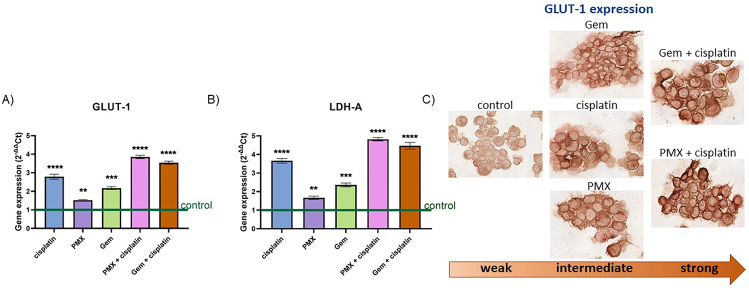
**Glucose transporter type 1 (GLUT-1) and lactate dehydrogenase A (LDH-A) expression in pleural MM.** GLUT-1 (A) and LDH-A (B) gene expression and immunocytochemical analysis of GLUT-1 (C) in pleural MM cells H2052 after 24 h of treatment with cisplatin, pemetrexed (PMX) and gemcitabine (GEM). Results are presented as mean ± SEM; ∗p < 0.05; ∗∗p < 0.01, ∗∗∗p < 0.005, ∗∗∗∗p < 0.0001 compared to control; one-way ANOVA with Holm-Sidak post hoc test.

To check whether the standard anticancer treatments can also affect the GLUT-1 expression in *in vitro* models, pleural MM cells (H2052) were treated for 24 h with compounds alone and combinations of GEM, cisplatin, and PMX. Gene expression of GLUT-1 and LDH-A was significantly higher after incubation with cisplatin, GEM, PMX and the combination of cisplatin with PMX and GEM, similar to changes noticed in patient tissue (Figure 2A,B). Since the gene expression analyses of LDH-A and GLUT-1 showed a similar trend, we selected GLUT-1 for protein-level validation by immunocytochemistry. We observed a relatively higher expression of GLUT-1 after a single treatment, while the effect of the combination of cisplatin with GEM or PMX was even more pronounced (Figure 2C).

The above results prompted us to check if inhibition of GLUT-1 and LDH-A would affect the mesothelioma cells.

### 3.4 Migration inhibition after treatment with the GLUT-1 and LDH-A inhibitors in mesothelioma cells

**Figure 3:**
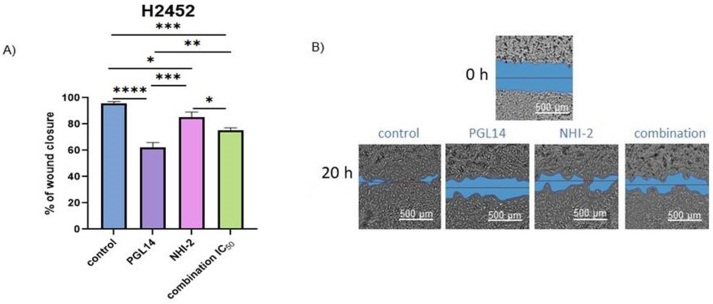
**Migration inhibition** after treatment with the PGL14 and NHI-2 in pleural mesothelioma (H2452) cell line (A) with representative images of the scratch (B) at 0 and 20 h. Results are presented as mean ± SEM; ∗p < 0.05, ∗∗p < 0.01, ∗∗∗p < 0.005, ∗∗∗∗p < 0.0001; one-way ANOVA with Holm-Sidak post hoc test.

Evaluation of mesothelioma cell ability to migrate after GLUT-1 and LDH-A inhibition was assessed by a wound healing assay. The progress of wound closure was monitored by capturing images at 0, 4, 8, 20 and 24 h after scratch making and adding the inhibitors. The results showed a significant decrease in the migration of H2452 cells after incubation with GLUT-1 and LDH-A inhibitors, with the most significant reduction seen with PGL14 (Figure 3). A similar pattern of changes was observed in the H2052 cells and peritoneal primary mesothelioma cells, STO and MESO-II (Supplementary Fig. S1).

### 3.5 Apoptosis induction and reactive oxygen species production increase in mesothelioma cells after combination treatment of GLUT-1 and LDH-A inhibitors

**Figure 4:**
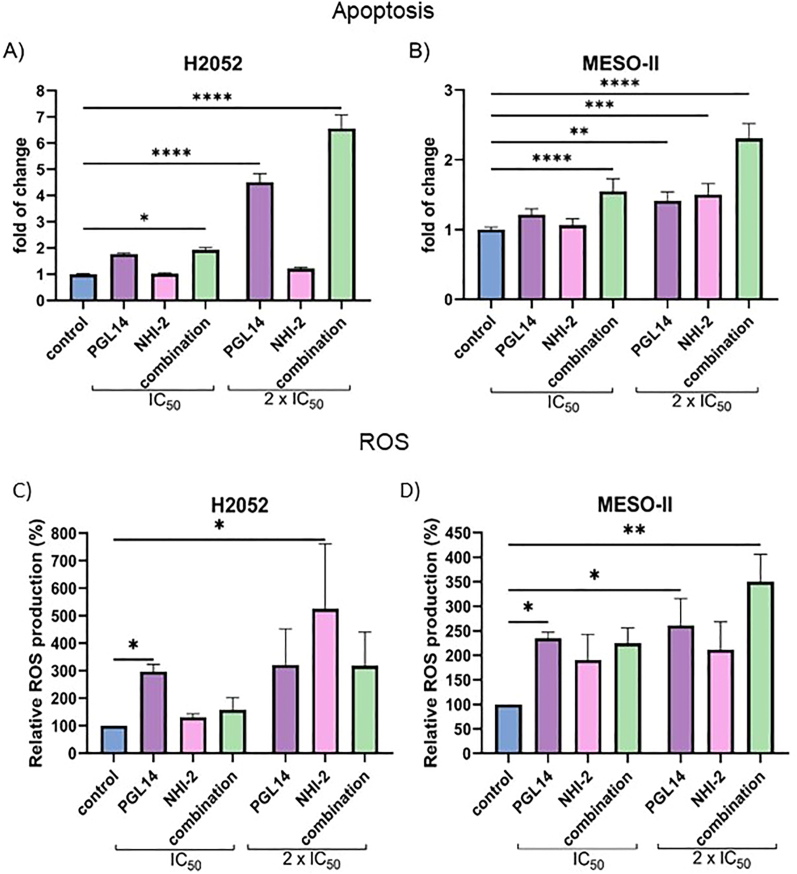
**Apoptosis induction and reactive oxygen species (ROS)** production after 48 h incubation with the IC_50_ concentration of PGL14 and NHI-2 in pleural and peritoneal mesothelioma cells. Apoptosis in pleural (A) and peritoneal (B) MM. ROS production in pleural (C) and peritoneal (D) MM cells. Results are presented as mean ± SEM; ∗p < 0.05; ∗∗p < 0.01, ∗∗∗p < 0.005, ∗∗∗∗p < 0.0001 compared to control; one-way ANOVA with Sidak's or Kruskal–Wallis test. Inhibitors: LDH-A (NHI-2) and GLUT-1 (PGL14).

To analyze the potential of GLUT-1 and LDH-A inhibitors to induce ROS production and apoptosis, mesothelioma cells were treated with the inhibitors for 48 h. In both pleural and peritoneal mesothelioma, the combination treatment led to a relevant increase in apoptosis at IC_50_ and 2-times IC_50_ concentrations. This effect was more pronounced in H2052 cells compared to MESO-II. On the contrary, only a single treatment with PGL14 at 2 × IC_50_ concentration resulted in the stimulation of apoptosis in both cell lines, but for NHI-2 only in MESO-II cells (Figure 4A,B).

PGL14 treatment led to a 3 times higher ROS production in H2052 and MESO-II cells. This effect was observed both at IC_50_ and 2 × IC_50_. However, LDH-A inhibition increased ROS production only in H2052 at 2 × IC_50_. Additionally, combination treatment led to a similar effect as for a single treatment, but in MESO-II at 2 × IC_50_ a relevant increase of ROS was observed, which was higher than each inhibitor separately (Figure 4C,D).

### 3.6 Spheroids disintegration occurred after LDH-A and GLUT-1 inhibition

**Figure 5:**
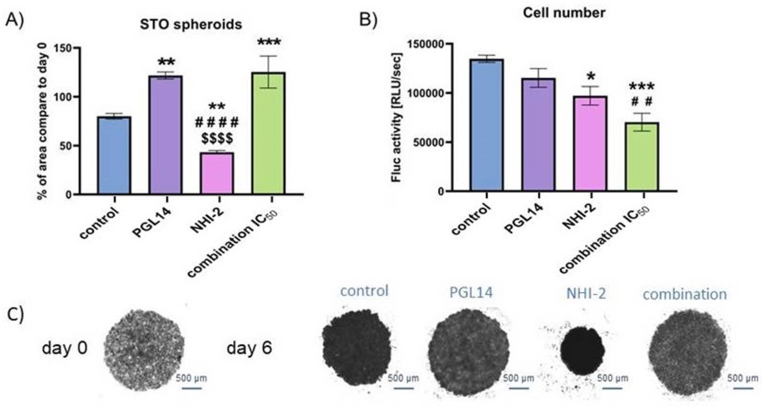
**Assessment of spheroids treated with the IC_50_ concentration of PGL14 and NHI-2 in STO cells.** (A) Changes in spheroid size at day 6 compared to day 0. (B) Number of cells forming the spheroids. Representative images of spheroids at day 0 and day 6 for each treatment group are reported in the bottom panel (C). Results are presented as mean ± SEM; n = 6; ∗p < 0.05; ∗∗p < 0.01, ∗∗∗p < 0.005, ∗∗∗∗p < 0.0001 compared to control; ####p < 0.0001 PGL14 compared to NHI-2, $$$$ p < 0.0001 NHI-2 compared to combination and ##p < 0.01 NHI-2 compared to combination (one-way ANOVA with Holm-Sidak post hoc test). Inhibitors of LDH-A (NHI-2) and GLUT-1 (PGL14).

To examine how compounds affect tumors' heterogeneous structure, we created 3-dimensional spheroids using mesothelioma cells. These spheroids were treated with IC_50_ concentrations of inhibitors every 2 days for 6 days. The changes in spheroids’ size were measured every 2 days. In the STO cell line at day 6, we observed a significant increase in spheroid size after PGL14 and combination treatment (Figure 5A). However, a visible disintegration of the spheroids was noticed both after GLUT-1 inhibition and combination treatment (Fig. 5C). Interestingly, LDH-A inhibition led to the shrinking of spheroids. The number of cells creating spheroids at the endpoint was measured using bioluminescence (Supplementary Fig. S2) and a reduction of cell numbers was observed after NHI-2 treatment and combination therapy (Fig. 5B). In pleural MM cell lines, we also observed an enlargement of spheroids after combination treatment with noticeable loose density (Supplementary Fig. S3).

## 4 Discussion

In this study, we conducted a comprehensive analysis of LDH-A and GLUT-1 expression in pleural MM, demonstrating a correlation between these key glycolysis effectors and clinical outcomes in a relatively large cohort of patients with this rare disease, which accounts for approximately 0.2% of all cancers [27]. Patients with both high LDH-A and GLUT-1 levels had the worst prognosis, suggesting that multiple mechanisms affecting glycolysis play key roles in tumor aggressiveness, as suggested by previous studies [28].

Additionally, we are the first to measure GLUT-1 and LDH-A expression in pleural MM tissues of patients before and after treatment with PMX/platinum regimens. We analyzed the expression of GLUT-1 and LDH-A in patients treated with PMX and platinum analogs, as this combination is the first-line treatment for MM (13). Interestingly, 45% of the patients exhibited elevated GLUT-1 levels (11 out of 24), while 55% did not show any change. The finding that patients with increased GLUT-1 expression had a worse prognosis suggests that cells adopting a more glycolytic phenotype after treatment may become more aggressive and chemoresistant.

As for LDH-A, the minimal changes observed post-treatment in MM tissues could be due to the already high pre-treatment expression of LDH-A in over 50% of samples. In contrast, our *in vitro* study showed a significant increase in LDH-A expression following PMX and cisplatin treatment, indicating the need for further investigation into LDH-A modulation and its impact, ideally in a larger cohort of samples for future studies.

The increased levels of GLUT-1 in nearly 50% of patients were linked to a significantly shorter survival, strongly supporting the role of this transporter in mediating chemotherapy resistance. Possibly, the increase in GLUT-1 is due to the heightened demand for glucose by cancer cells to counteract the effects of chemotherapy. This aligns with the use of PET as a metabolic imaging modality for oncological assessment, including re-staging and treatment monitoring of MM [29]. The increased accumulation of the radiotracer fluorodeoxyglucose (FDG), labeled with fluorine-18 ([^18^F]), is primarily due to the elevated expression of glucose transporters, especially GLUT-1, along with the upregulation of key glycolytic enzymes such as hexokinase and the concurrent downregulation of gluconeogenic enzymes.

Mesothelioma is characterized by a high degree of inter-patient heterogeneity, with histological subtype being the main driver of diversity. In our series, no distinct pattern of LDH-A or GLUT-1 expression emerged within the small subgroup of non-epithelioid patients. We fully acknowledge that the limited number of non-epithelioid tumors included in our study precludes drawing definitive conclusions regarding potential differences according to histological subtype. However, non-epithelioid patients are currently treated with immunotherapy in routine clinical practice. Therefore, future prospective trials investigating metabolic markers such as LDH-A and GLUT-1 should ideally focus on epithelioid patients to ensure a more homogeneous study population and allow for more robust conclusions.

Several regulatory mechanisms ensure that GLUT-1 expression is finely tuned in response to both internal and external cellular environments, playing a critical role in cellular metabolism and adaptation. In many tumor types, GLUT-1 expression is upregulated by various factors, including the stabilization of hypoxia-inducible factor-1 under low oxygen conditions. Oncogenic stimuli from *MYC* and *RAS*, along with abnormal modulation of signaling pathways such as PI3K/AKT and mTOR, also contribute to the increased expression of GLUT-1 [30].

Previous studies have demonstrated that cisplatin-resistant tumors frequently exhibit constitutive activation of mTORC1 signaling [31]. Similarly, PMX-resistant MM cell lines, where the expression of PMX-targeted enzymes was not elevated, showed increased levels of phosphorylated AKT [32]. Additionally, we recently observed upregulation of phospho-AKT in cells resistant to GEM [33].

In line with these findings, our current study reveals that in pleural MM cells, the expression of GLUT-1 and LDH-A significantly increases following standard treatment with cisplatin, PMX, and GEM. These results led us to explore whether inhibiting GLUT-1 and LDH-A could impact MM cell behavior, potentially suggesting new therapeutic strategies.

In this study, we compared the effects of GLUT-1 and LDH-A inhibitors on both pleural and peritoneal mesothelioma cells. While recognizing that these subtypes can exhibit distinct biological behaviors and treatment responses, our aim was to capture the complexity of the disease and identify potential therapeutic strategies that may be effective across different MM subtypes. In addition, our earlier findings indicated that pleural MM exhibited a higher ATP/ADP ratio and lower glycolysis compared to peritoneal MM [14], underscoring the need for a comprehensive approach.

The varying responses observed in pleural MM cells may, at least in part, be related to differences in the expression levels of LDH-A and GLUT-1. Although both cell lines exhibit very high expression of these markers, H2452 appears to show comparatively higher levels (Fig. S4). This difference may potentially contribute to the greater sensitivity of H2452 cells to the inhibitors, as suggested by lower IC_50_ values and more pronounced spheroid disruption compared to H2052 cells.

Remarkably, in the present study, we found that inhibiting GLUT-1 had a stronger effect on cell migration compared to the combined treatment, except for the MESO-II cell line. However, the combination of GLUT-1 and LDH-A inhibition resulted in a significant increase in cell death in both pleural and peritoneal MM. Additionally, ROS production was higher with the combination treatment, particularly in peritoneal MM. Most importantly, in the 3D mesothelioma spheroid model, which more accurately simulates the tumor microenvironment, the combination treatment disrupted spheroid structure in all cell lines and significantly reduced cell numbers in STO spheroids. This indicates that the combined treatment's effects are more prominent in a 3D model, which is crucial for understanding tumor behavior in a clinical context.

Though we do not have a definitive explanation for why simultaneous inhibition did not further influence migration beyond what was observed with the single treatment, we can hypothesize that the activity of the single compounds may have already reached a plateau. Nonetheless, our results are consistent with a previous study by Mogi et al. who reported reduced cell invasion in pleural MM cells (CRL-5915) after treatment with GLUT-1 siRNA [34]. Similarly, LDH-A inhibition resulted in decreased MM cell migration, likely due to high extracellular acidification caused by elevated LDH-A activity and lactate production, which promotes cell invasion [2]. Migration inhibition was also observed in a previous study following LDH-A knockdown and treatment with FX-11, an LDH-A inhibitor, in gastric cancer cells [35].

As for functional data, in our previous study, we observed a significant reduction in intracellular nucleotide levels in peritoneal MM following the combined treatment, whereas the reduction was minor with single treatment. Furthermore, the combination index analysis revealed a synergistic effect of PGL14 and NHI-2 in both pleural and peritoneal MM [14].

Inhibiting GLUT-1 and LDH-A also resulted in increased apoptosis and elevated ROS levels, especially when these inhibitors were used in combination or at higher concentrations. The higher induction of apoptosis and ROS observed in pleural MM compared to peritoneal MM can be connected with a significantly higher impact of inhibitors on glycolysis and mitochondria in pleural than peritoneal MM, as we described in our previous paper [14]. LDH downregulation can induce apoptosis in cancer cells through elevated ROS levels and increased Bax expression, preceded by a reduction in mitochondrial membrane potential, which aligns with our findings [36]. Recent research has also demonstrated that 1-(phenylseleno)-4-(trifluoromethyl) benzene (PSTMB), a novel LDH-A inhibitor, similarly induces apoptosis and ROS production in HT29 colon cancer cells [37]. The low ROS production observed after LDH-A inhibition may seem unexpected, as blocking LDH-A (i.e., pyruvate-to-lactate conversion) should redirect pyruvate toward mitochondrial oxidation, theoretically increasing ROS levels. However, our results are consistent with a study on Galloflavin, another LDH inhibitor, which exhibited an apoptotic effect in pancreatic cancer cells but only in co-culture with pancreatic stellate cells [38]. Additionally, in our previous experiments, we assessed mitochondrial respiration in pleural MM after treatment with these inhibitors and did not observe significant changes, suggesting a minor impact on mitochondrial ROS production [14].

Additionally, several studies have documented an apoptotic effect resulting from GLUT-1 inhibition, consistent with our findings. For instance, the specific GLUT-1 inhibitor WZB117 activated the AMPK pathway, leading to apoptosis in human breast carcinoma cells, including the adriamycin-resistant MCF7/ADR cell line, and also resensitized these cells to adriamycin [39]. Conversely, the study by Oh et al. demonstrated that downregulation of GLUT-1 in breast cancer cells (MDA-MB-231 and Hs578T) led to increased resistance to doxorubicin, GEM, irinotecan, etoposide, and paclitaxel through modulation of the Akt/GSK-3β/β-catenin/survivin signaling pathway [40]. These conflicting results may be attributed to differences in cell models and the specific experimental conditions, underscoring the need for further research to elucidate the specific mechanisms underlying apoptosis induction in various tumor types.

An important model that bridges preclinical studies to more clinically relevant settings is the use of spheroids, which avoid the high costs of animal models and adhere to the 3R principles (Replacement, Reduction, Refinement). Consequently, we chose to use this 3D model, as it more accurately mimics the clinical characteristics of tumors and offers better predictions of the clinical response to newly tested drugs compared to traditional monolayer cultures [41]. In these experiments, we observed that PGL14 and combination treatments led to the disruption of the spheroid structure. Additionally, the number of cells forming spheroids decreased following incubation with NHI-2 and the combination treatment. This is in agreement with a previous study showing that LDH-A inhibition by NHI-2, when conjugated with glucose, significantly disrupted pancreatic cancer spheroids and enhanced the effectiveness of GEM [42].

In summary, our study highlights the potential of targeting GLUT-1 and LDH-A for MM treatment. Elevated expression of LDH-A and GLUT-1, along with increased GLUT-1 levels in tumors following chemotherapy, suggests their role in MM resistance. This resistance represents a significant challenge for conventional treatments, underscoring the need for novel therapeutic strategies.

Pharmacological studies reveal that PGL14 and NHI-2 effectively inhibit migration of pleural and peritoneal MM cells, increase apoptosis and ROS levels, with a more pronounced effect observed when both inhibitors are used in combination. These results align with our previous findings on the impact of these inhibitors on energy metabolism in MM cells [14]. Notably, spheroid culture experiments, which more closely mimic clinical conditions, further confirm the efficacy of GLUT-1 and LDH-A inhibition. Despite minor differences, the MM cells exhibited a similar response pattern to the novel LDH-A and GLUT-1 inhibitors, including pleural MM cells derived from epithelioid (H2052) and biphasic (H2452) histotypes, as well as primary peritoneal mesothelioma cells. Based on these observations, we believe that our results remain reliable, even though not all available cell lines could be used in every analysis.

Taking into account that GLUT-1 expression increases after standard chemotherapy, novel inhibitors of LDH-A and GLUT-1 could be considered as a potential second-line treatment option for patients with MM. However, these inhibitors may be particularly relevant in clinical settings involving epithelioid-type pleural MM and peritoneal MM, whereas immunotherapy has demonstrated efficacy in non-epithelioid pleural MM.

In conclusion, there is an urgent need to elucidate the precise mechanisms underlying MM resistance. This study represents a significant step forward in this endeavor, highlighting the potential of targeting LDH-A and GLUT-1 as promising strategies for developing new treatments for this aggressive cancer.

## CRediT authorship contribution statement

**Marika A. Franczak:** Writing – original draft, Visualization, Methodology, Investigation, Funding acquisition, Formal analysis, Data curation, Conceptualization. **Federica Borea:** Writing – original draft, Visualization, Investigation, Formal analysis, Conceptualization. **Valentina Donati:** Resources, Funding acquisition, Formal analysis, Data curation, Conceptualization. **Marta Aliprandi:** Writing – original draft, Visualization, Resources, Methodology, Investigation, Formal analysis, Conceptualization. **Amir Avan:** Methodology, Investigation, Formal analysis, Conceptualization. **Valentina Doldi:** Resources, Methodology, Formal analysis, Data curation, Conceptualization. **Giulia Bononi:** Resources, Investigation, Data curation. **Kin Yip Tam:** Project administration, Investigation, Data curation. **Carlotta Granchi:** Resources, Project administration, Data curation. **Ryszard T. Smolenski:** Project administration, Funding acquisition, Data curation. **Paolo A. Zucali:** Project administration, Funding acquisition, Data curation. **Filippo Minutolo:** Resources, Project administration, Funding acquisition, Data curation. **Godefridus J. Peters:** Writing – review & editing, Supervision, Project administration, Funding acquisition, Data curation. **Elisa Giovannetti:** Writing – review & editing, Supervision, Funding acquisition, Data curation.

## Financial support and sponsorship

This research was partially supported by ‘the Law Offices of Peter G. Angelos Grant’ from the Mesothelioma Applied Research Foundation (MARF), United States (EG, PAZ, FM and GJP), Fondazione Humanitas, Milano, Italy (EG, PAZ, FB, and MA), 10.13039/501100005010Italian Association for Cancer Research, AIRC/Start-Up, Italy (EG), CCA Foundation grants 2013 and 2015, The Netherlands (EG and GJP), National Science Center of Poland (2018/31/B/NZ7/02909, RTS, GJP, MAF; 2023/49/N/NZ7/01893 MAF, GJP), a young investigator grant from the Purine and Pyrimidine Society (MAF), KWF Dutch Cancer Society (EG). The research leading to these results has also received funding from the 10.13039/501100000780European Union - NextGenerationEU through the Italian Ministry of University and Research under PNRR - M4C2-I1.3 Project PE_00000019 ″HEAL ITALIA” to Filippo Minutolo, CUP I53C22001440006. The views and opinions expressed are those of the authors only and do not necessarily reflect those of the European Union or the European Commission. Neither the European Union nor the European Commission can be held responsible for them.

## Declaration of competing interest

The authors declare that they have no known competing financial interests or personal relationships that could have appeared to influence the work reported in this paper.

## Data Availability

Data will be made available on request.
