# Supplementary material for: Dynamic evaluation of the glycolytic determinants LDH-A and GLUT-1 enhances prognostic significance and their inhibition affects the growth of mesothelioma spheroids
Source: Mol Metab. 2026 Mar 23;107:102356. doi: 10.1016/j.molmet.2026.102356 (PMC13084315; doi:10.1016/j.molmet.2026.102356)
Supplement: Multimedia component 1 [file mmc1.docx]

Supplementary Material

**Table S1. The growth inhibitory concentration (IC_50_) of GLUT-1 (PGL14) and LDH-A (NHI-2) inhibitors.**

| Inhibitor | **H2052** | **H2452** | **MESO-II** | **STO** |
| --- | --- | --- | --- | --- |
| **PGL14 [µM]** | 44.4 ± 2.9 | 32.6 ± 1.3 | 6.4 ± 0.2 | 45.9 ± 4.3 |
| **NHI-2 [µM]** | 43.5 ± 4.7 | 25.0 ± 2.7 | 18.5 ± 2.0 | 24.7 ± 2.6 |

Malignant mesothelioma cell lines treated for 72 h in normoxia. Results are presented as mean ± SEM; the experiments were performed at least in triplicate and repeated three times [1].


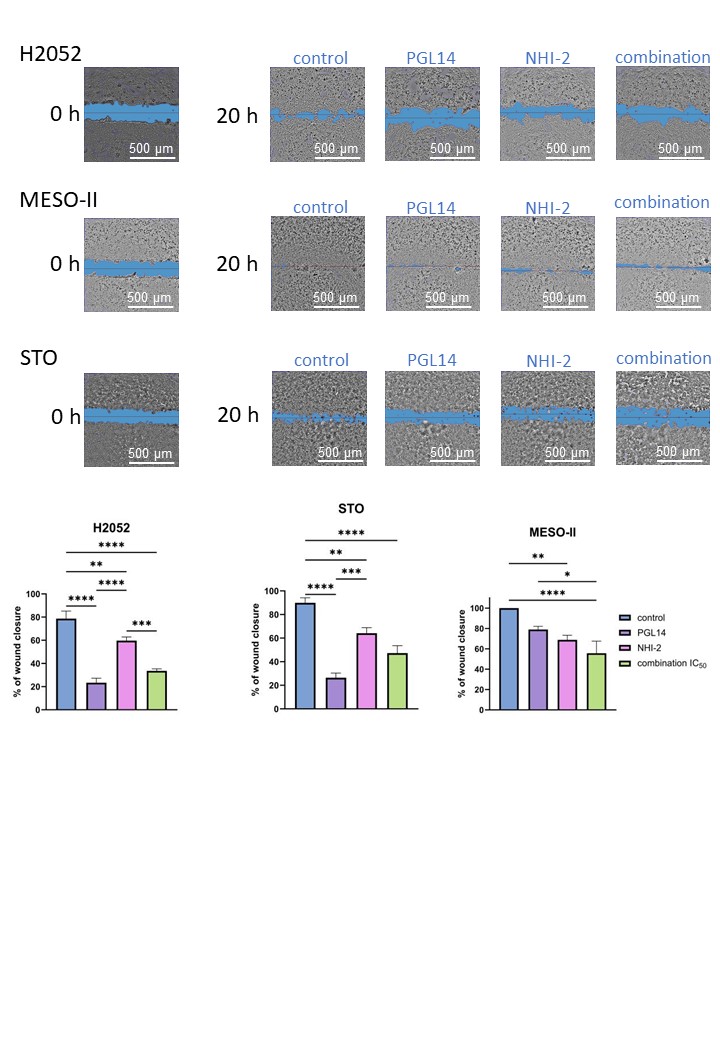


**Figure S1.** Migration inhibition after 20 h of treatment with the PGL14 and NHI-2 in primary (STO, MESO-II) and immortalized (H2052) mesothelioma cells. Results are presented as mean ± SEM.
* p<0.05; ** p<0.01, *** p<0.005, **** p<0.0001; one-way ANOVA with Sidak’s test. Inhibitors: LDH-A (NHI-2) and GLUT-1 (PGL14).


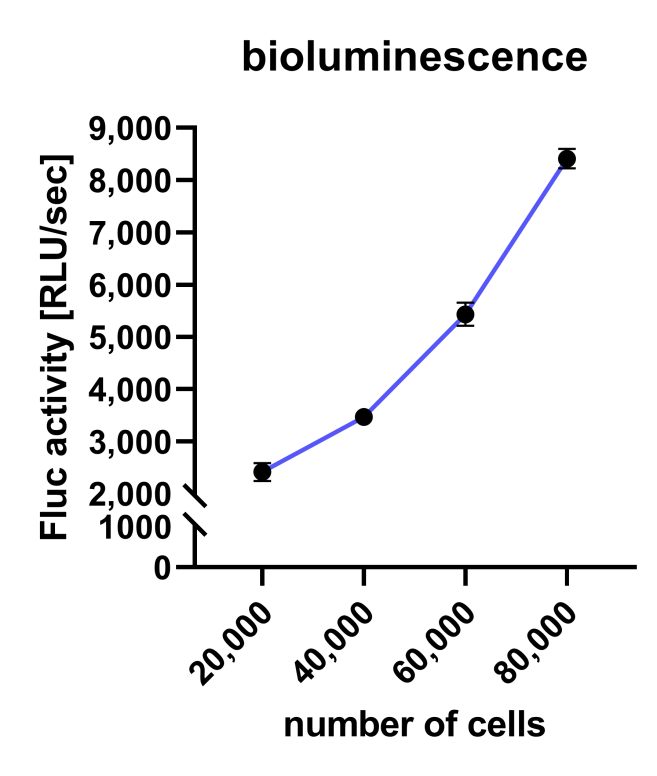


**Figure S2.** Bioluminescence assay results showing proportion between Fluc activity signal with STO cell number. Results are presented as mean ± SEM.


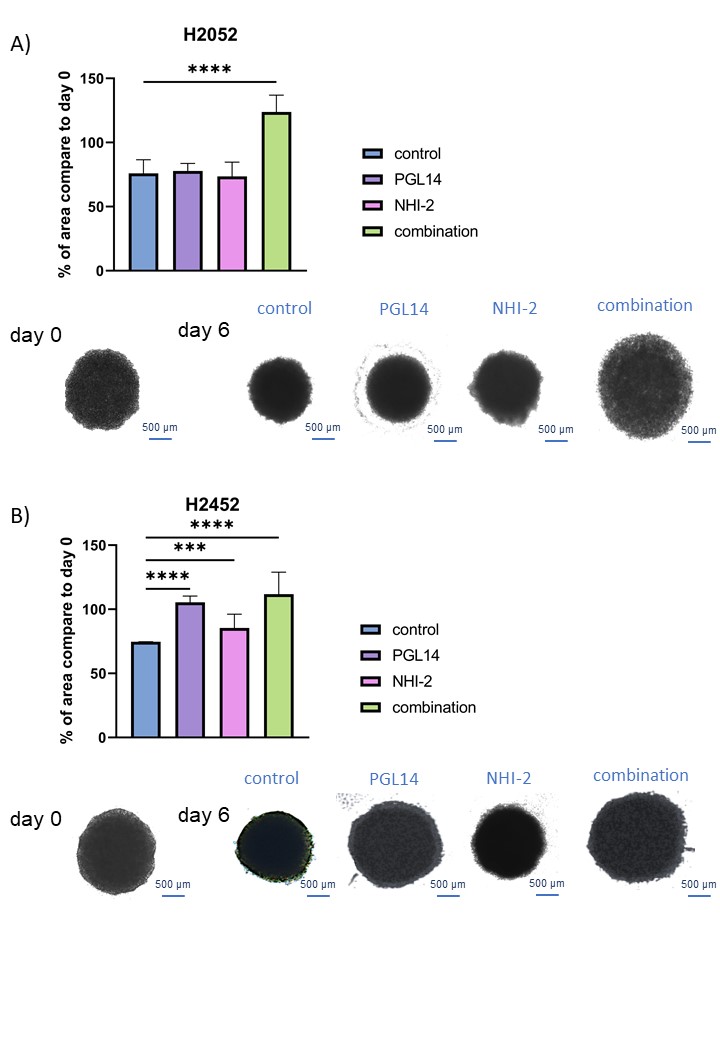


**Figure S3.** Spheroids treatment with the IC_50_ concentration of PGL14 and NHI-2 in pleural mesothelioma cell lines. Changes of spheroids size at 6 days compared to day 0 in H2052 (A) and H2452 (B) cells. Results are presented as mean ± SEM; *** p<0.005, **** p<0.0001 compared to control; one-way ANOVA with Holm-Sidak post hoc test. Inhibitors: LDH-A (NHI-2) and GLUT-1 (PGL14).


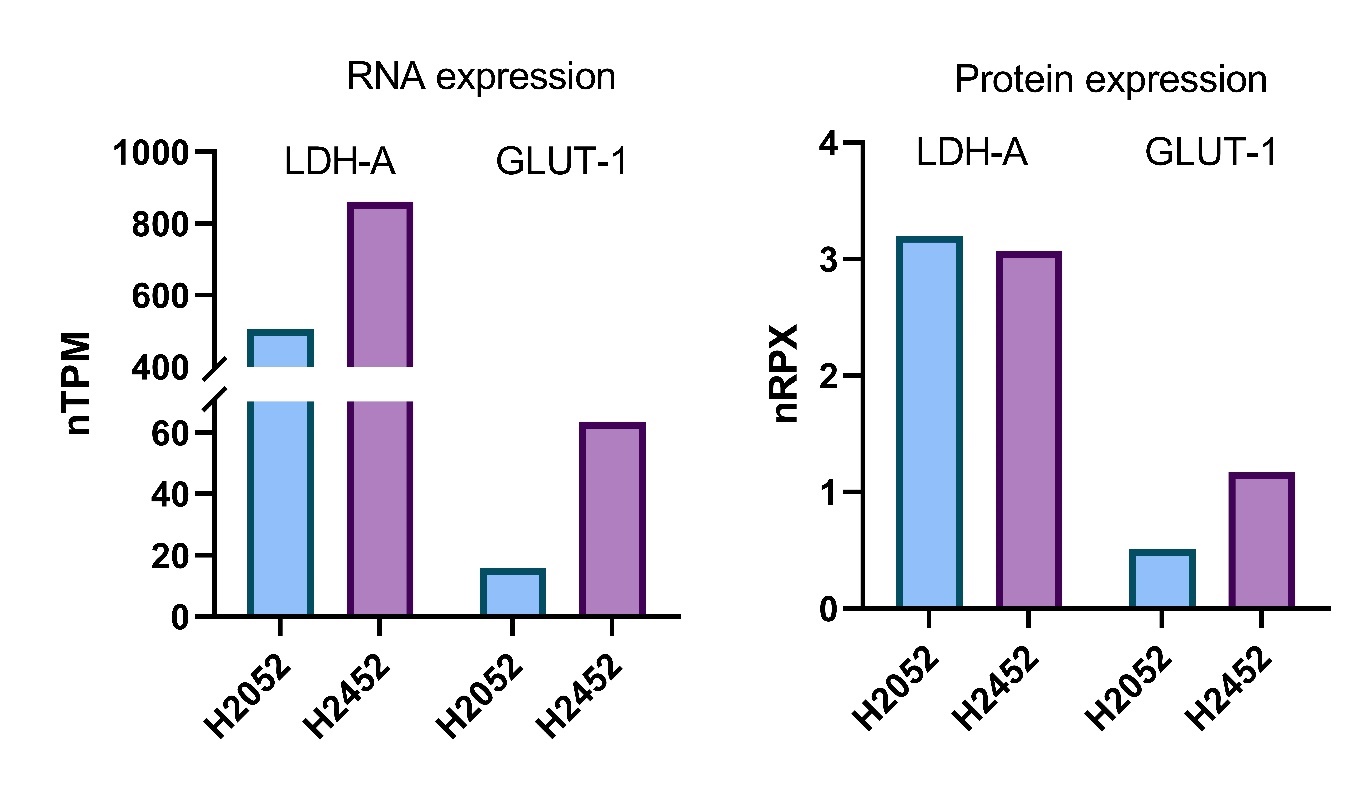


Figure S4. RNA and protein expression of lactate dehydrogenase A (LDH-A) and glucose transporter 1 (GLUT-1, SLC2A1 gene) in malignant pleural mesothelioma cell lines (H2052, H2452). Data from the Human Protein Atlas [2]. nTPM normalized transcripts per million; nRPX normalized relative protein expression.

**References**

[1] Franczak, M.A., Krol, O., Harasim, G., Jedrzejewska, A., Zaffaroni, N., Granchi, C., et al., 2023. Metabolic Effects of New Glucose Transporter (GLUT-1) and Lactate Dehydrogenase-A (LDH-A) Inhibitors against Chemoresistant Malignant Mesothelioma. International Journal of Molecular Sciences 24(9): 7771, Doi: 10.3390/ijms24097771.

[2] the Human Protein Atlas. https://www.proteinatlas.org/.
